# Nodules de lisch dans la neurofibromatose type 1

**DOI:** 10.11604/pamj.2017.27.218.11517

**Published:** 2017-07-21

**Authors:** Yassine Abaloun, Yousra Ajhoun

**Affiliations:** 1 Université Mohammed V Souissi, Service d’Ophtalmologie de l’Hôpital Militaire Mohamed V, Hay Riad, Rabat, Maroc

**Keywords:** Nodules de lisch, neurobiromatose 1, maladie de Von Recklinghausen, Lisch nodules, neurofibromatosis type 1, Von Recklinghausen disease

## Abstract

Neurofibromatosis type 1 (NF1) or Von Recklinghausen disease manifests as cutaneous café-au-lait spots and neurofibromas. It is one of the most common autosomal dominant genetic diseases. It is extremely variable in its individual manifestation. Cutaneous and neurologic symptoms are the most common manifestations but it can also affect other organs including eyes, bones and other areas. Lisch nodules are the most common ocular manifestations in NF-1. They are asymptomatic small pigmented iris tumors (iris hamartomas) which can help suggest the diagnosis of NF1 as they are characteristic of this disease and mostly occur in adult patients. We report the case of a 45-year old female patient followed for a neurofibromatosis type 1 retained on the presence of multiple cutaneous café-au-lait spots and neurofibromas. Ophthalmologic examination showed visual acuity of 10/10 P3 in both eyes. Biomicroscopic examination showed Lisch nodules of the iris in both eyes (A,B).

La neurofibromatose 1 (NF1) ou maladie de Von Recklinghausen est une maladie qui se manifeste par des taches café au lait cutanées et des neurofibromes. C'est une des maladies génétiques les plus fréquentes à transmission autosomique dominant. Ses manifestations sont extrêmement variables d'un malade à l'autre. Les manifestations les plus souvent rencontrées sont cutanées et neurologiques mais d'autres organes peuvent être touchés comme l'œil, les os…Les nodules de Lisch constituent la manifestation oculaire la plus fréquente de la NF1 et correspondent à des petites tumeurs pigmentées de l'iris (hamartomes iriens) qui n'entraînent aucun symptôme mais sont une aide au diagnostic car ils sont caractéristiques de la maladie et présents chez la plupart des malades adultes. Nous rapportons le cas d'une patiente de 45 ans suivie pour une neurofibromatose type 1 retenue sur la présence de multiples taches cutanées café au lait et des neurofibormes. L'examen ophtalmologiques trouve une acuité visuelle à 10/10 P3 en ODG avec à l'examen bio-microscopique des nodules de lisch iriens au niveau des deux yeux (A,B).

**Figure 1:**
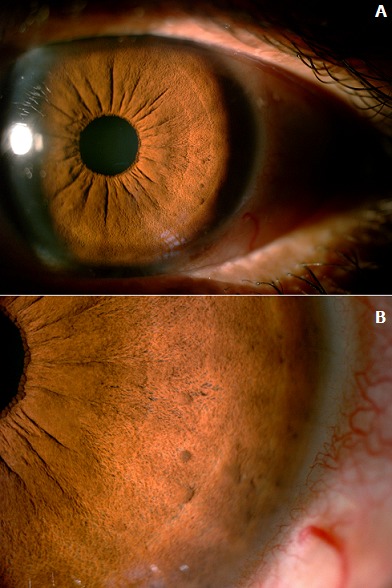
A) segment antérieur de l’œil gauche montrant les nodules iriens de lisch; B) segment antérieur montrant les nodules de lisch sous forme de lésions d’aspect gélatineux, surélevés en « dôme » et à bord net

